# Supplementary material for: MetaRibo-Seq measures translation in microbiomes
Source: Nat Commun. 2020 Jun 29;11:3268. doi: 10.1038/s41467-020-17081-z (PMC7324362; doi:10.1038/s41467-020-17081-z)
Supplement: Supplementary file 10 — Supplementary Data 7 [file 41467_2020_17081_MOESM10_ESM.zip › File2/Confidence_VeryHigh_Taxonomy/192014_out.krona.html]

Javascript must be enabled to view this page.

members
magnitude
magnitudeUnassigned
count
unassigned
taxon
rank

192014\_out

4

2
superkingdom
4

4
phylum
1239

4
class
186801

186802
order
4

1
family
31979

1649459
1
genus


SRS147766\_contig\_number\_10110
154046
1
species

186803
2

SRS077730\_contig\_number\_contig-100\_2665.45317
family
1

2302969

SRS053214\_contig\_number\_contig-100\_19933.19934
species
1

541000
1
family

genus
1
1263

165186

SRS103987\_contig\_number\_contig-100\_10747.10747
1
species
